# Large Laryngeal Lipoma with Extra Laryngeal Component Mimics Mixed Form Laryngocele: A Case Report

**DOI:** 10.22114/ajem.v0i0.196

**Published:** 2019-07-28

**Authors:** Ahmad Rezaee Azandaryani, Mohamadmehdi Eftekharian, Mehrdad Taghipour

**Affiliations:** 1. Department of Radiology, Hamadan University of Medical Sciences, Besat Hospital, Hamadan, Iran.; 2. Department of General Surgery, Hamadan University of Medical Sciences, Besat Hospital, Hamadan, Iran.

**Keywords:** Case reports, Head and neck neoplasms, Laryngeal neoplasms, Lipoma

## Abstract

**Introduction:**

Lipomas are the most common benign neoplasms, occurring in any part of the body where fat is present. Their occurrence in the head and neck is not common. Here, we report a large laryngeal lipoma with extra laryngeal component, mimicking mixed form of laryngocele.

**Case presentation:**

A 47-year-old man presented with a 3-year history of hoarseness, intermittent dyspnea and mass sensation in the neck. The patient was submitted to indirect laryngoscopy; a large submucosal mass obliterating the left side of the supraglottic larynx and partially obstructing the airway was found. Enhanced computed tomography (CT) scan demonstrated non-enhancing homogeneous hypodense fat density mass lesion measured 55*45*32 mm, extending through the thyroid membrane to parapharyngeal space and showing extra laryngeal component with an intact laryngeal mucosa lesion. Open surgery of the submucosal mass was performed. Pathology examination confirmed the diagnosis of lipoma.

**Conclusion:**

Neck lipomas are also typically asymptomatic, but can compress nearby structures, causing symptoms such as hoarseness, dyspnea and dysphagia. When symptomatic, they should be removed via surgery.

## Introduction

Lipomas are slow growing, well circumscribed and benign tumors, which is one of the most common benign neoplasms, appearing in any part of the body, where fat is present. Lipomas are more commonly found in males and their peak incidence is in the fourth and fifth decade of life (1). They occur commonly on the extremities, which are areas of high subcutaneous fat. Their occurrence in the head and neck is not common, and approximately 15% of lipomas are found in this region (1, 2). Posterior cervical triangle is the most common location of the neck, which is involved, and it comprises about 0.6% of benign neoplasms of the larynx and hypopharynx. Lipomas in the neck are typically asymptomatic, but can compress nearby structures, causing symptoms such as hoarseness, dyspnea and dysphagia (2–5). Here, we report a large laryngeal lipoma with an extra laryngeal component, mimicking mixed form of laryngocele.

## Case presentation

**Figure 1:**
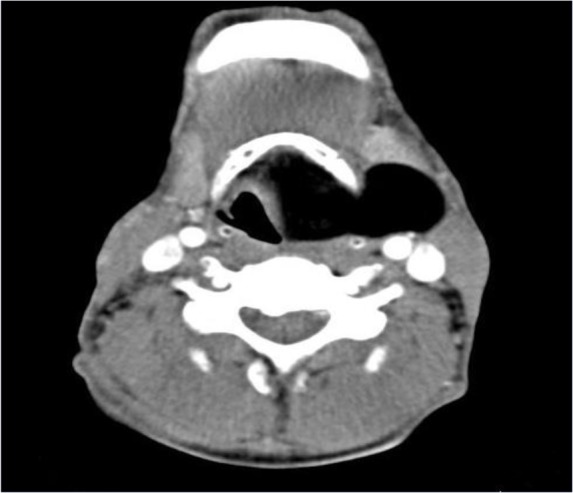
Axial soft tissue window image shows homogenous hypodense mass lesion in the left supraglottic area, extending to parapharyngeal space resembling a large laryngocele

**Figure 2:**
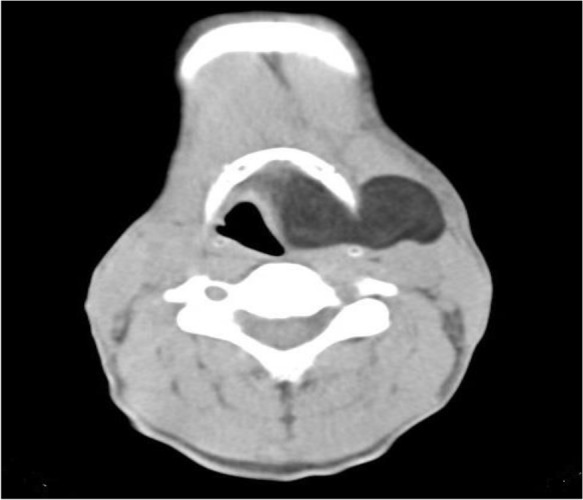
Axial lung window image demonstrated non-enhancing homogenous hypodense fat density mass lesion (average density -86 HU) measured 55*45*32 mm, extending through the thyroid membrane to parapharyngeal space

**Figure 3:**
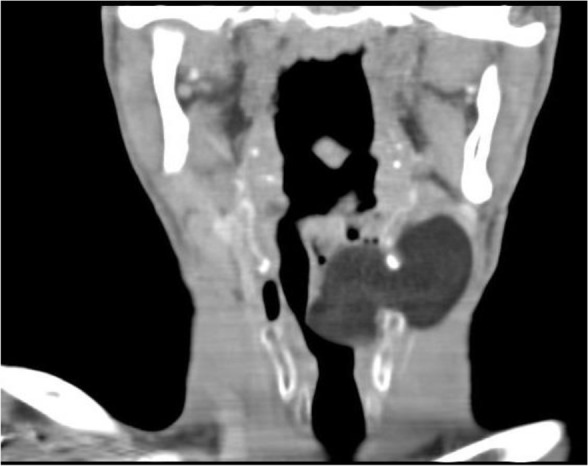
Reconstructed coronal lung window image

A 47-year-old man presented with a 3-year history of hoarseness, intermittent dyspnea and mass sensation in the neck. He had no special past medical history, smoking or alcohol abuse. In physical examination, a soft mass in the left cervical region near trachea was palpable. The patient was submitted to indirect laryngoscopy, and a large submucosal mass obliterating the left side of the supraglottic larynx and partially obstructing the airway was found. At the next step, endoscopy showed a large mass arising from the left supraglottic and glottic region. Contrast enhanced computed tomography (CT) scan images demonstrated non-enhancing homogeneous hypodense fat density mass lesion (average density was −86 HU) measured 55*45*32 mm, extending through the thyroid membrane to parapharyngeal space, and showing an extra laryngeal component with an intact laryngeal mucosa lesion, appearing as hypodense as air and mimicking a large laryngocele in paranchymal window (Figure 1). In the lung window, image differentiation of lesion density and air is completely visualized (Figure 2, 3). Open surgery of the submucosal mass was performed. Pathology examination revealed an eight-centimeter encapsulated tumor containing uniform, mature adipocytes at microscopic exam, and confirming the diagnosis of lipoma.

The patient was discharged the day after excision of the lipoma. He was symptom-free upon discharge. He was followed up in the clinic after two months and again at six months, and reported complete resolution of his symptoms.

## Discussion

Lipomas could be categorized into extrinsic and intrinsic types. Extrinsic lipomas, arising from the posterior surface of arytenoid, lingual surface of the epiglottis and pharyngeal wall, were more common than true intrinsic laryngeal lipomas (77% versus 23%). This is due to the presence of small amounts of adipose tissue in the larynx, found at the aryepiglottic fold, false cords and epiglottis. Lipoma occurs much less frequently in true vocal cords, which has a scant amount of fat tissue (4, 6). In our case, both intrinsic and extrinsic components were appeared. Intrinsic component originated from aryepiglottic fold and supraglottic region, extending through the thyroid membrane to parapharyngeal space and showed an extra laryngeal component with an intact laryngeal mucosa. The location of the lipoma can affect signs and symptoms. Commonly, the symptom of sensation of a mass in the throat is present, as well as dysphagia or dyspnea in larger lesions. If the lesion is an intrinsic type, it can directly influence the vocal cord function, leading to hoarseness and voice change. Our case had three years’ history of symptoms, which was aggravated gradually. Our case represents both intrinsic and extrinsic components, causing symptoms of hoarseness, intermittent dyspnea, and sensation of a mass in the throat.

CT scan has a role in terms of evaluating the size and extent of the tumor. Laryngeal lipomas appear frequently as a single hypodense non- enhancing mass, with density ranging from −60 to −120 Hounsfield Unit (HU). Magnetic resonance imaging (MRI), if performed, would help for exact demarcation. It shows a high intensity lesion on T1 weighted images and T2 weighted fast spin echo sequences (6). Surgical excision, whether endoscopic or open, is the preferred treatment of symptomatic lipomas in the head and neck, for minimizing the recurrence chance (7).

## Conclusions

Neck lipomas are also typically asymptomatic, but can compress nearby structures, causing symptoms such as hoarseness, dyspnea and dysphagia. When symptomatic, they should be removed via surgery.
